# From Family Patterns to Eating Disorder Risk: The Role of Social Media, Appearance Ideals, and Body Image Among Emerging and Young Adults

**DOI:** 10.3390/nu18101497

**Published:** 2026-05-08

**Authors:** Lior Gendelman, Ora Peleg, Efrat Hadar

**Affiliations:** Departments of Education, School Counseling, The Max Stern Yezreel Valley College, Afula 193060088, Emek Yezreel, Israel or orap@yvc.ac.il (O.P.); or ehadar@edu.haifa.ac.il (E.H.)

**Keywords:** differentiation of self, problematic social media use, internalization of appearance ideals, negative body image, risk of eating disorders

## Abstract

**Background:** Eating disorders are on the rise among emerging and young adults, driven by familial dynamics, social media, psychological traits, and cultural, genetic, and peer factors. Yet not all underlying variables are fully understood. This study explores relationships between the differentiation of self (a crucial family pattern), problematic social media use, internalization of appearance ideals, negative body image, and the risk of eating disorders, aiming also to identify risk profiles. An additional objective was to examine differences between emerging adults and young adults, two age groups that have recently experienced increased prevalence of eating disorders. **Methods:** Participants included 333 emerging (*n* = 114, 34.2%) and young (*n* = 219, 65.8%) adults aged 18–40 (mean age 29.53; 207 females, 126 males) who completed questionnaires assessing the study variables. **Results:** The results revealed a sequential mediation effect: differentiation of self contributed to risk of eating disorders via problematic social media use, internalization of appearance ideals, and negative body image. Emerging adults (18–25) reported a higher risk of eating disorders, problematic social media use, internalization of appearance ideals, and emotional reactivity/fusion with others than young adults (26–40). A risk profile emerged: young, single, child-free women with a lower education and socioeconomic status, lower differentiation of self, and higher internalization of appearance ideals and problematic social media use. **Conclusions:** These findings highlight the importance of addressing both familial and societal factors—particularly differentiation of self, problematic social media use, and internalization of appearance ideals—in prevention and intervention programs for eating disorders. Developing tailored strategies for high-risk groups, such as young, single women with lower education and socioeconomic status, may enhance program effectiveness.

## 1. Introduction

The prevalence of eating disorders (EDs) has been rising globally among adolescents and emerging and young adults in recent years [1,2], making it imperative to consider the factors that increase this risk. EDs are common mental health conditions affecting approximately 10% of the global population, with a marked increase in cases since the COVID-19 pandemic. About 75–90% of those affected are female, with higher prevalence traditionally observed among adolescent girls and young women in Western countries, although rates are increasing among males and children [3]. In Israel, available estimates remain partial. According to a report by the Israeli Knesset Research and Information Center [4], based on Ministry of Health data, approximately 6–8% of females aged 15–24 are estimated to suffer from EDs. Broader estimates suggest that between 69,000 and 97,000 adolescents and young adults may be affected; however, these figures exclude males and other age groups and therefore likely underestimate the true prevalence.

The tripartite influence model [5] posits that pressures from family, friends, and the media contribute to the internalization of appearance ideals, ultimately leading to body dissatisfaction. Recent research underscores the model’s relevance, highlighting the significant role of social media in exacerbating body dissatisfaction [6] and increasing the risk of EDs [7]. Furthermore, studies also suggest that problematic social media use, internalization of appearance ideals, and family dynamics—particularly differentiation of self (DoS)—play a critical role in elevating the risk of EDs [8]. However, although problematic social media use and differentiation of self (DoS), have each been linked to ED risk, less is known about how these factors operate together within a single integrative model. A deeper exploration of these interconnected factors is essential for bridging gaps in understanding how familial dynamics and social media uniquely interplay to drive the rising prevalence and growing risk of EDs. Therefore, the main objective of this study is to investigate the key factors contributing to the risk of EDs, focusing on the mediating roles of problematic social media use, internalization of appearance ideals, and negative body image in the relationship between DoS and the risk of EDs.

Most research on the risk of EDs has primarily focused on adolescents, with limited attention given to emerging and young adults aged 18–40. Given the importance of these developmental stages, the present study aims to deepen the understanding of the factors contributing to EDs within these later age groups. Thus, we examine gender and age differences between emerging and young adults with the aim of establishing a comprehensive profile of risk factors for individuals prone to developing EDs.

### 1.1. Differentiation of Self

The concept of DoS, introduced in family systems theory [9], emphasizes the balance between two opposing forces: (a) individuality, driving independence, and (b) togetherness, promoting emotional closeness. These forces are an integral part of the intergenerational transmission of emotional and behavioral patterns, shaping emotional processes within the family [10,11]. DoS includes four dimensions. Emotional reactivity refers to the intensity of emotional responses to stressful events. I-position describes the ability to stay true to one’s values and needs, even when not aligned with those of significant others. Emotional cutoff describes a tendency to detach physically, verbally, or emotionally from others to regulate emotions. Finally, fusion with others reflects the tendency to form dependent relationships and constantly seek acceptance from others [10]. Research has shown gender differences in these dimensions, with women exhibiting higher emotional reactivity and fusion with others, while men display higher emotional cutoff [11,12].

Bowen [9] pointed to the impact of DoS on mental and physical health, which has been supported by recent studies, highlighting its crucial role in promoting mental well-being [13] and physical health [14]. Research indicates that DoS significantly shapes thinking and decision-making processes, coping with stress, and the internalization of appearance ideals [6]. Furthermore, studies have revealed significant associations between low DoS and negative body image [15], as well as the risk of EDs [8]. Individuals with low DoS are particularly vulnerable to external pressures, including social media influences [14,15]. They often rely heavily on others and experience elevated anxiety when their emotional needs are unmet—patterns commonly associated with problematic social media use as a coping mechanism [16,17].

### 1.2. Problematic Social Media Use and Internalization of Appearance Ideals

Social media serves as a primary platform for interpersonal communication and information sharing [18], particularly among emerging and young adults [19,20]. In Israel, social media use increased from 65% in 2015 to 86% in 2020, with higher usage among females and among emerging and young adults [21].

While social media fosters connectivity, it also contributes to problematic use, characterized by excessive online engagement and neglect of daily activities [22,23]. Problematic social media use has been linked to negative psychological, social, and physical consequences, even though it is not classified as an addiction in the DSM-5-TR [24,25]. A major concern is the role of social media in perpetuating unrealistic beauty standards through constant exposure to tailored images of celebrities, influencers, and peers, fostering a culture that emphasizes external appearance and shapes perceptions of “ideal” body structure [26]. This exposure drives the internalization of appearance ideals (the unconscious adoption of societal standards of beauty), shaping self-perceptions and contributing to body dissatisfaction [27]. Research has demonstrated that this internalization process serves as a crucial mediating mechanism, specifically linking problematic social media use to negative body image outcomes [28]. Objectification theory [29] explains how societal and media influences shape self-perception, emphasizing how women’s physical appearance is often treated as an object of evaluation, exacerbating feelings of inadequacy and negative body image [30]. There are also some gender differences: while women are more likely to internalize thin ideals, men face pressure to achieve muscularity [31,32]. Moreover, younger adults tend to internalize these ideals at higher rates than older age groups [33].

There is evidence that problematic social media use is significantly associated with negative body image and an increased risk of EDs [34]. Similarly, higher levels of appearance ideal internalization are consistently linked to a greater risk of developing EDs [31,35].

### 1.3. Negative Body Image

Body image is a multidimensional concept encompassing thoughts, feelings, and behaviors involving one’s physical appearance [36,37]. Discrepancies between actual and desired appearance may lead to a negative body image [38,39], a common issue among emerging and young adults, especially women [30,40]. Research has identified several factors contributing to these negative perceptions, including low levels of DoS [15], problematic social media use [32], and internalization of appearance ideals [28]. Negative body image carries significant implications for both mental and physical health [40,41] and is a well-documented risk factor for EDs [7,42,43].

### 1.4. Risk of Eating Disorders

According to the DSM-5-TR, EDs are severe mental health conditions characterized by persistent disturbances in eating behaviors and distorted body image, posing significant risks to mental and physical health [24]. These conditions have the highest mortality rates among mental disorders [44]. The DSM-5-TR identifies three main types: Anorexia Nervosa, Bulimia Nervosa, and Binge Eating Disorder [24].

The prevalence of EDs has risen globally in recent decades, particularly in Western societies, ranging from 0.1% to 3.8% [45,46]. Social media use and family dynamics, particularly low DoS [2], as well as additional social, psychological, biological, and cultural aspects, have been identified as risk factors [47,48]. Specifically, research has demonstrated significant associations between increased risk of EDs and lower levels of DoS [8], as well as between increased risk of EDs and higher levels of problematic social media use [34], internalization of appearance ideals [31,35], and negative body image [7,43]. While EDs affect diverse populations, they are most prevalent among adolescent girls [46,49]. In recent years, however, prevalence has increased among young women and, to a lesser extent, among young men [50,51].

### 1.5. Emerging and Young Adulthood

The current study focused on emerging adults (18–25) and young adults (26–40). Emerging adulthood is a developmental stage defined as the transition from adolescence to full adulthood [52]. It represents a distinct developmental period characterized by identity exploration, feelings of being “in-between,” instability, self-focus, and a sense of possibilities. Both emerging and young adulthood are characterized by ongoing development, challenges related to identity formation, and the negotiation of key life domains such as relationships, career paths, and financial independence [53]. During these stages, individuals often transition into more stable adult roles, such as career development, long-term relationships, and family responsibilities, while enhancing their decision-making skills and developing an increased ability to cope with stress and interpersonal conflicts [7].

However, these stages differ in the degree of identity consolidation and life stability. Emerging adulthood is marked by greater instability, ongoing identity exploration, uncertainty regarding career paths, and continued (often partial) dependence on family support. In contrast, young adulthood is typically characterized by greater role stability and a more consolidated sense of self, although it remains a period of continued development and life challenges [54,55].

Importantly, these differences may shape individuals’ susceptibility to social influences, body image concerns, and ED risk. While both stages offer many opportunities, emerging and young adults also face unique vulnerabilities, including psychological distress due to uncertainty in relationships, careers, and housing [52]. Emerging and young adults are particularly engaged with social media [56,57]. For instance, in Israel, their usage rates more than double those of older adults [19]. As mentioned, this heightened involvement can negatively affect body image [32] and contributes to a higher risk of EDs [52].

Recent studies reveal worldwide ED prevalence rates of 5.5–17.9% among young women and 0.6–2.4% among young men [49]. Despite this rising prevalence, research focusing on the unique vulnerabilities and contributing factors among young adults remains scarce, reinforcing the critical need for further investigation to address this growing public health concern. Accordingly, the focus on emerging and young adults reflects the relevance of these two developmentally distinct stages to questions of identity, social influence, body image, and ED risk.

### 1.6. The Present Study

Research has highlighted the significant role of social media in shaping appearance pressures and increasing the risk of EDs [58]. Specifically, the internalization of appearance ideals has been shown to mediate the relationship between problematic social media use and negative body image [28]. However, susceptibility to social media influences is not uniform and varies according to individual and contextual factors such as age, gender, and culture [59]. In this context, lower DoS, reflecting deficits in emotional regulation and the ability to maintain clear interpersonal boundaries, may represent a key vulnerability factor. Individuals with lower DoS tend to exhibit heightened sensitivity to external influences and a stronger reliance on social approval [11], which may increase their susceptibility to the pressures embedded in social media environments characterized by intensified social comparison and external reinforcement processes [7]. This heightened susceptibility may, in turn, facilitate the internalization of appearance ideals, thereby contributing to negative body image and increasing the risk of EDs. Beyond its role in shaping vulnerability to sociocultural influences, DoS—as a central family based construct—has also been linked to cognitive and emotional processes, including decision-making, coping with stress, and identity formation [6], as well as to ED risk. Taken together, these findings underscore the importance of examining DoS alongside sociocultural factors within an integrative framework to better understand the mechanisms underlying ED risk.

Despite the increasing prevalence of EDs among young and emerging adults, research on this population remains scarce. Building on the convergence between family systems theory—particularly the construct of DoS, which has been shown to contribute to mental well-being [13] and is negatively associated with negative body image [15] and the risk of EDs [8]—and the tripartite influence model, which emphasizes the impact of peers, parents, and media on the internalization of appearance ideals [5], and given existing knowledge gaps and the critical role of family patterns, the present study examines the complex interplay of differentiation of self, problematic social media use, internalization of appearance ideals, and the risk of eating disorders among emerging and young adults.

In light of the limited availability of comprehensive epidemiological data—both globally and within specific contexts such as Israel—and despite accumulating evidence indicating a marked increase in ED prevalence in recent years, advancing research in this domain constitutes an urgent public health priority.

By addressing an understudied population, this research provides valuable insights into the mechanisms driving EDs, offering a deeper understanding of the factors contributing to this growing public health concern. The study also examines group differences among male and female emerging adults and young adults, as well as individuals scoring above the clinical cutoff on the EAT-26 versus those who do not, with the aim of delineating a comprehensive profile of those at heightened risk of developing EDs.

### 1.7. Research Hypotheses

**Figure 1:**
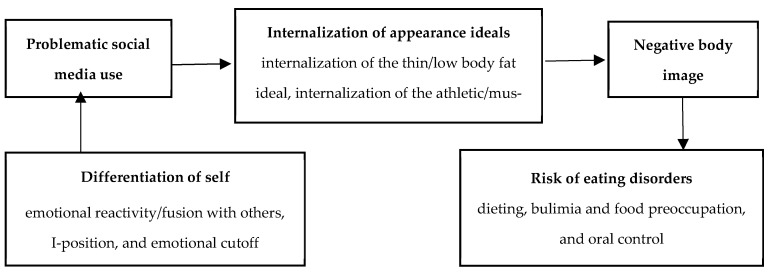
The mediation model.

1. The relationship between DoS (emotional reactivity, I-position, emotional cutoff, and fusion with others) and the risk of EDs (dieting, bulimia and food preoccupation, and oral control) will be serially mediated through the following variables: problematic social media use, internalization of appearance ideals (including internalization of the thin/low body fat ideal, internalization of the athletic/muscular ideal, family pressure, peer pressure, and media pressure), and negative body image (see Figure 1). Specifically, the following:
  - Problematic social media use will mediate the relationship between DoS and the internalization of appearance ideals.
  - Internalization of appearance ideals will mediate the relationship between problematic social media use and negative body image.
  - Negative body image will mediate the relationship between internalization of appearance ideals and the risk of EDs.
2. Age differences will be found, with emerging adults (18–25) reporting higher problematic social media use, greater internalization of appearance ideals, higher negative body image, and higher levels of risk of EDs than young adults (26–40).
3. Gender differences will be found, with women reporting higher levels of emotional reactivity/fusion with others, internalization of appearance ideals, negative body image, and the risk of EDs, while men will report a higher level of emotional cutoff and internalization of the athletic/muscular ideal.
4. Clinical differences will be found, with participants scoring above the clinical cutoff on the EAT-26 showing lower DoS, higher problematic social media use, greater internalization of appearance ideals, and more negative body image than those not meeting clinical criteria.

## 2. Method

### 2.1. Participants

The participants included 333 Israeli emerging (*n* = 114, mean age = 21.33, SD = 2.26, range = 18–25) and young (*n* = 219, mean age = 33.79, SD = 4.57, range = 26–40) adults (mean age = 29.53, *SD* = 7.11, range = 18–40), recruited through convenience sampling using two recruitment channels: advertisements posted on social media and distribution through an online survey company. Participants were recruited from across Israel. Eligibility criteria included being between the ages of 18 and 40, living in Israel, and being able to complete a self-report questionnaire in Hebrew. Exclusion criteria included participants who were not sufficiently proficient in Hebrew to understand the questionnaire instructions, those who provided incomplete responses, and those who did not meet the age criteria. The sample included 60% females, with 55% married or in a committed relationship, and one third being parents. Two thirds of participants had parents who were married or in a committed relationship, and one fifth had parents who were divorced. Most participants were Jewish (99%) and secular (70%), with 55% holding academic degrees. Approximately half reported below-average economic status (*n* = 89, 26.8%), and a quarter reported above-average economic status (*n* = 87, 26.1%). About half (*n* = 171, 51.4%) reported normal BMI values, some reported underweight (*n* = 24, 7.2%), and others reported overweight (*n* = 86, 25.8%) or obesity (*n* = 52, 15.6%).

### 2.2. Instruments

**A demographic questionnaire** was constructed for the current research. It inquired about age, marital status, and self-reported height and weight (for calculating BMI).

**Differentiation of Self Inventory—Revised** (DSI-R, [60,61]; Hebrew version, [62,63]) is a self-report questionnaire that assesses relationships with significant others and an individual’s ability to balance cognitions and emotions. It contains 46 items across four subscales: emotional reactivity, I-position, emotional cutoff and fusion with others. Participants rated items on a 6-point Likert scale from 1 (completely untrue) to 6 (completely true). Sample item: “I am very sensitive to criticism” (emotional reactivity). Subscale scores were averaged. Higher DoS is indicated by lower scores on emotional reactivity, emotional cutoff, and fusion with others, and by higher scores on I-position. Due to a high correlation between emotional reactivity and fusion with others (*r* = 0.78, *p* < 0.001), their combined mean score was calculated (α = 0.92). In prior validation analyses, these subscales demonstrated a high degree of correlation, leading to the recommendation that they be combined (14). Given their strong association in the present sample, they were combined into a single index reflecting difficulties in relational self-regulation. The current study showed good reliability: emotional reactivity/fusion with others, α = 0.92; I-position, α = 0.84; and emotional cutoff, α = 0.84.

**Social Media Disorder Scale** (SMD, [64]; Hebrew version, [65]). The SMD is a nine-item scale examining problematic social media use. Participants assigned the dichotomous items a 1 (*no*) or 2 (*yes*), with problematic use indicated by six or more “yes” responses. Sample item: “You often felt dissatisfied because you wanted to spend more time on social media.” Total scores range from 0 to 9, summing all items. Reliability was acceptable in the current study: α = 0.70.

**Sociocultural Attitudes Towards Appearance Questionnaire-4** (SATAQ-4; [66]); Hebrew version created and back-translated for the current study). This scale examines appearance ideals and social pressures regarding appearance. It contains 22 items across 5 subscales: internalization of the thin/low body fat ideal, internalization of the athletic/muscular ideal, family pressure, peer pressure, and media pressure. Sample item: “I feel pressure from the media to lower my body fat level” (pressure from media). Participants rated items on a 5-point Likert scale from 1 (strongly disagree) to 5 (strongly agree). Subscale scores were averaged. In the current study reliability was high for all scales: thin/low body fat ideal, α = 0.88; athletic/muscular ideal, α = 0.91; family pressure, α = 0.88; peer pressure, α = 0.89; and media pressure, α = 0.95. Inter-scale correlations ranged from *r* = 0.14 (*p* = 0.011) to *r* = 0.53 (*p* < 0.001), with the total score showing excellent reliability: α = 0.91.

**The Body Shape Questionnaire C-8** (BSQ-C8, [67]; Hebrew version created and back-translated for the current study) is a shortened version of the original BSQ [68], assessing body image over the last four weeks. It includes eight items; sample item: “Have you felt excessively large and round?” Participants rated items on a 6-point Likert scale from 1 (never) to 6 (always). Scores range from 34 to 204, with higher scores indicating more negative body image; 98 is the clinical cutoff. Reliability in the current study was high: α = 0.91.

**The Eating Attitudes Test-26** (EAT-26, [69,70]; Hebrew version, [71]) examines the risk of EDs. It includes 26 items across three subscales: dieting, bulimia and food preoccupation, and oral control. Sample item: “I feel very guilty after eating” (dieting). Participants rated items on a 6-point Likert scale from 0 (never) to 5 (always). Total scores of 20 or higher indicate a risk of EDs. Due to a strong correlation in the current study between (a) dieting and (b) bulimia and food preoccupation, the total score was used (α = 0.89). In the present study reliability was α = 0.88 for dieting, α = 0.80 for bulimia and food preoccupation, and α = 0.60 for oral control (relatively lower reliability).

### 2.3. Procedure

The research was approved by the Ethics Committee of a northern Israeli college (Approval No. YVC EMEK 2024-52). Participants received a Google Forms questionnaire link via social media or a survey company. Before completing the questionnaire, they signed an informed consent form that assured anonymity and indicated that data would be used solely for research and academic purposes. They were also advised that participation was voluntary and could be discontinued at any stage. Completing the questionnaire took approximately 15 min. The questionnaires began with a description of the study’s objectives followed by an informed consent form. The questionnaires were then presented within the online form in the same sequence as described in the Instruments section.

### 2.4. Data Analysis

Data were analyzed by SPSS (Version 27.0) [72] and AMOS (version 29). There were no missing values in the data, and descriptive statistics and internal consistencies were calculated. Several variables (problematic social media use, negative body image, and risk of EDs) were positively skewed (skewness = 0.78 to 1.75, *SE* = 0.13) which violated normality assumptions and could have biased the statistical models of the study. They were thus log-transformed (resulting skewness: 0.44 to 0, *SE* = 0.13). Economic status (ordinal, skewness = 0.12, *SE* = 0.13) was treated as continuous. Pearson correlations, analyses of variance and *t*-tests examined relationships between study variables and demographics, particularly for the risk of EDs. A repeated-measures ANOVA examined the dimensions of internalization of appearance ideals and gender interaction, using estimated marginal means. Logistic regressions assessed associations between the study variables and risk of ED classification.

Path analysis examined the study model, using Chi square, NFI, TLI, CFI, RMSEA and SRMR as fit measures. Analyses controlled for gender, age, economic status, and BMI, noting significant correlations among control and independent variables. Serial mediation was tested using bootstrapping (5000 samples, bias-corrected 95% confidence interval) with standardized variables. The standardization makes the coefficients comparable across different scales, and allows us to determine relative coefficient strength. Gender differences were examined through linear regression models with gender interactions interpreted using simple slope analysis [73], as sample size precluded full model interactions or separate gender models.

## 3. Results

### 3.1. Descriptive Results

The distribution of study variables showed moderate–low to low means. Clinical range classifications included 10% (*n* = 35) for problematic social media use, 35% (*n* = 116) for negative body image, and 15% (*n* = 51) for risk of EDs. Study variables were significantly intercorrelated in the expected directions, with positive correlations among emotional reactivity/fusion, emotional cutoff, problematic social media use, internalization of appearance ideals, and negative body image, while I-position was negatively associated with these variables (see Table 1).

### 3.2. Preliminary Analyses: Associations Between Study Variables and Demographic Variables

Age was negatively associated with most study variables (except for I-position) (*r* = −0.11, *p* = 0.049 to *r* = −0.31, *p* < 0.001), indicating that younger participants reported lower DoS, higher problematic social media use, greater internalization of appearance ideals, a lower body image, and higher risk of EDs. Similar associations were found for non-married/non-relationship status, having no children, and lower education level, which were significantly correlated (*r* = 0.22, *p* < 0.001 to *r* = 0.55, *p* < 0.001). Further, the sample was divided into two age groups: emerging adulthood (18–25, *n* = 114, 34.2%) and young adulthood (26–40, *n* = 219, 65.8%). A series of *t*-tests was run to examine differences in the study variables between these groups. Results showed that emerging adults reported higher risk of EDs, *t*(331) = 5.74, *p* < 0.001, more problematic social media use, *t*(331) = 4.42, *p* < 0.001, greater internalization of appearance ideals, *t*(331) = 3.92, *p* < 0.001, higher emotional reactivity/fusion with others, *t*(331) = 5.64, *p* < 0.001, and higher emotional cutoff, *t*(331) = 3.53, *p* < 0.001, than young adults.

Moreover, economic status was negatively associated with emotional cutoff (*r* = −0.11, *p* = 0.045), body image (*r* = −0.16, *p* = 0.003), and the risk of EDs (*r* = −0.15, *p* = 0.007). Finally, BMI was positively associated with a higher internalization of appearance ideals (*r* = 0.20 *p* < 0.001) and a lower body image (*r* = 0.34, *p* < 0.001). Demographic differences were also found regarding the risk of EDs, with higher risk among females, *t*(309.09) = 3.90, *p* < 0.001, emerging adults, *t*(331) = 5.74, *p* < 0.001, single individuals, *t*(325) = 4.27, *p* < 0.001, non-parents, *t*(331) = 3.85, *p* < 0.001, those with less than a college education, *t*(331) = 2.91, *p* = 0.004, and those with below-average economic status, *F*(2, 330) = 5.37, *p* < 0.001. No differences were found in terms of religiosity, parents’ marital status, or BMI.

#### Gender Differences

Significant gender differences were found across multiple study variables, necessitating gender control in the main analysis of the research model. Females showed higher levels of emotional reactivity/fusion with others, *t*(331) = 7.47, *p* < 0.001, problematic social media use, *t*(222.79) = 2.84, *p* = 0.005, internalization of appearance ideals, *t*(331) = 2.64, *p* = 0.009, negative body image, *t*(331) = 5.05, *p* < 0.001, and risk of EDs, *t*(309.09) = 3.90, *p* < 0.001 than males. No gender differences emerged for emotional cutoff or I-position.

Further analysis of internalization of appearance ideals yielded additional gender differences. Analysis of the means (Table 2) revealed that internalization of the thin/low body fat ideal, *t*(302.83) = 3.60, *p* < 0.001, and media pressure, *t*(317.93) = 8.26, *p* < 0.001, was higher among females, while internalization of the athletic/muscular ideal, *t*(331) = −2.07, *p* = 0.039, and peer pressure, *t*(230.90) = −2.56, *p* = 0.011, was higher among males. Rank ordering these dimensions by gender revealed a significant interaction effect, *F*(4, 1324) = 32.13, *p* < 0.001, η^2^ = 0.088. For males, internalization of the thin/low body fat and athletic/muscular ideals ranked highest, followed by family pressure, media pressure, and peer pressure, *F*(4, 328) = 29.79, *p* < 0.001, η^2^ = 0.267. For females, internalization of the thin/low body fat ideal and media pressure ranked highest, followed by internalization of the athletic/muscular ideal, family pressure, and peer pressure, *F*(4, 328) = 140.25, *p* < 0.001, η^2^ = 0.631.

In contrast to the gender differences reported above, correlations within each gender showed similar patterns, with significant links between internalization of appearance ideals and other variables. Higher internalization of appearance ideals was associated with lower body image and greater risk of EDs. Higher emotional reactivity/fusion with others, emotional cutoff, and problematic social media use and lower I-position were generally linked to higher internalization of appearance ideals.

Given these observed gender differences, as well as the associations noted between other background variables (age, economic status, and BMI) and the study variables, the research model was examined while controlling for gender, age, economic status, and BMI.

### 3.3. The Study Model

**Figure 2:**
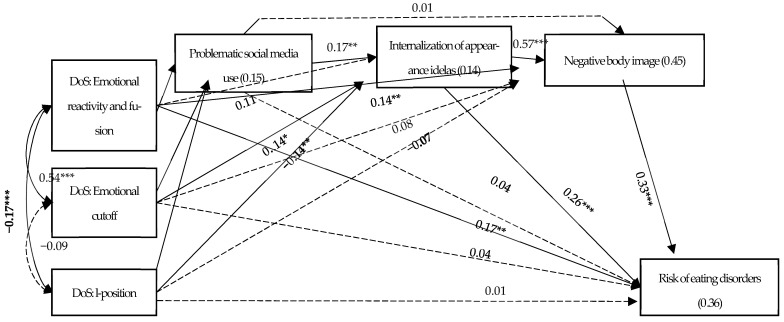
Serial mediation for the risk of eating disorders. Note. R^2^—values within rectangles. β—standardized regression coefficients—values above one-way arrows. r—correlations alongside two-way arrows. Solid arrows reflect significant results while dashed arrowes reflect non-significant results. * *p* < 0.05, ** *p* < 0.01, *** *p* < 0.001.

The study model was examined using path analysis, with the risk of EDs as the dependent variable and controlling for gender, age, economic status, and BMI. Intercorrelated DoS dimensions served as independent variables, with problematic social media use, internalization of appearance ideals, and negative body image as serial mediators. Internalization of appearance ideals was represented in the model by a composite mean calculated across the five SATAQ-4 subscales. Using standardized variables and correlating control variables, the serial mediation was tested with bootstrapping (5000 samples, bias-corrected 95% CI). The control variables are not shown. The model showed good fit: χ^2^(17) = 20.88, *p* = 0.232, *NFI* = 0.976, *TLI* = 0.985, *CFI* = 0.995, *RMSEA* = 0.026, and *SRMR* = 0.039 (see Figure 2 and Table 3).

The model revealed several significant direct and indirect associations among the study variables (*p* = 0.011 to *p* < 0.001). Higher emotional reactivity/fusion with others and emotional cutoff, and lower I-position were associated with greater problematic social media use, which in turn was linked to higher internalization of appearance ideals, negative body image, and increased risk of EDs. Additional direct associations included: higher emotional cutoff and lower I-position with internalization of appearance ideals, and higher emotional reactivity/fusion with others with both negative body image and risk of EDs. Significant indirect paths were observed (see Table 3), with the central serial mediation fully supported across all DoS dimensions (*p* = 0.011 to *p* < 0.001) and with partial mediation paths from problematic social media use and internalization of appearance ideals also significant (*p* = 0.001 and *p* < 0.001).

Additional significant indirect paths emerged (see Figure 2). Higher emotional cutoff and lower I-position were linked to greater internalization of appearance ideals, negative body image, and higher risk of EDs. Higher emotional reactivity/fusion with others was associated with poorer body image, contributing to increased risk of EDs. Higher emotional reactivity/fusion with others and emotional cutoff, and lower I-position, were associated with problematic social media use, which in turn was linked to higher internalization of appearance ideals and risk of EDs. Lastly, higher emotional cutoff and problematic social media use, and lower I-position, were directly associated with greater internalization of appearance ideals, leading to higher risk of EDs.

Finally, gender differences in the study model were examined. Due to the sample size (*n* = 126 males, and *n* = 207 females), rather than testing full model interactions or separate gender models, linear regression models with gender interactions were calculated. Two significant interactions emerged. First, the interaction between gender and problematic social media use was significant for negative body image (β = 0.15, *p* = 0.006), showing a significant positive association for males (effect = 0.06, *SE* = 0.03, *p* = 0.043, 95%CI = 0.01, 0.11), but a non-significant one for females (effect = −0.05, *SE* = 0.03, *p* = 0.096, 95%CI = −0.10, 0.01). Second, the interaction between gender and negative body image was significant for the risk of EDs (β = −0.15, *p* = 0.011), showing significant positive associations for both males (effect = 0.27, SE = 0.08, *p* < 0.001, 95%CI = 0.12, 0.42) and females (effect = 0.49, SE = 0.07, *p* < 0.001, 95%CI = 0.36, 0.63), with a stronger association for females (*p* = 0.047).

### 3.4. Eating Disorder Risk: The Cutoff Point

Using the cutoff point for risk of EDs (score = 20), 51 participants (15.3%) were classified at risk and 282 (84.7%) were not. Table 4 presents associations between study variables and risk of ED classification, controlling for gender, age, economic status, and BMI. Results show significant associations between most study variables and the risk of EDs: higher internalization of appearance ideals (internalization of the thin/low body fat ideal, internalization of the athletic/muscular ideal, family pressure, and media pressure), greater emotional reactivity/fusion with others, lower I-position, and lower body image were associated with positive risk of ED classification.

## 4. Discussion

The main objective of this study was to investigate the role of problematic social media use, internalization of appearance ideals and negative body image as mediators in the relationship between DoS and the risk of EDs. The study also examined differences between various groups: emerging adults versus young adults, males versus females, and participants scoring above the clinical cutoff on the EAT-26 versus those who did not. The findings support the proposed mediation model, shedding light on pathways to the risk of EDs by integrating familial patterns with psychosocial dimensions. They underscore the interconnected nature of these factors, suggesting that DoS is associated with the risk of EDs through problematic social media use, internalization of appearance ideals, and negative body image. The analysis also identified differences between research groups, providing a basis for profiling individuals at risk of developing EDs.

### 4.1. The Mediation Model

The fully supported serial mediation model (Hypothesis 1) provides significant clarity regarding the mechanisms linking DoS to the risk of EDs. First, problematic social media use was found to mediate the relationship between DoS and the internalization of appearance ideals (Hypothesis 1a). Consistent with prior research [16,17], it is suggested that individuals with low DoS may use social media as a means of avoiding emotional distress and dissatisfaction associated with unstable self-identity [13,74]. This tendency is associated with greater susceptibility to media content, which in turn is linked to stronger endorsement of appearance ideals and higher levels of their internalization. Second, the internalization of appearance ideals was found to mediate the relationship between problematic social media use and negative body image, confirming Hypothesis 1b and being consistent with the internalization of these ideals. Internalization is related to a discrepancy between perceived appearance and internalized standards [29,33], which in turn may be associated with negative body image and dissatisfaction with self-perception [25,29,33].

Finally, negative body image was found to mediate the relationship between internalized appearance ideals and the risk of EDs, corroborating Hypothesis 1c. Individuals who internalize unattainable ideals may feel inadequate and engage in unhealthy eating behaviors, such as restrictive dieting or binge eating, to align with these ideals [28,75]. These behaviors may increase the likelihood of developing EDs, underscoring the pivotal role of negative body image in this pathway [76,77].

Beyond the mediation model, several direct relationships emerged. Emotional reactivity/fusion with others was directly linked to negative body image. Individuals with low DoS may merge with others’ opinions [13], which is linked to greater pressure to please those close to them. This, in turn, could be associated with a more negative body image, particularly when individuals feel compelled to lose weight to meet others’ expectations. Low interpersonal DoS also corresponds with intrapersonal processes, including difficulties in emotional regulation and attempts to regulate emotions through eating [6]. Moreover, higher emotional cutoff and lower I-position were associated with greater internalization of appearance ideals, reflecting reliance on external standards and susceptibility to media influence [17]. Individuals with low I-position may struggle to differentiate their thoughts from those of others, increasing their vulnerability to media appearance ideals and problematic social media use [16].

Furthermore, internalization of appearance ideals was also directly linked to increased risk of EDs, as internalized standards may pressure individuals toward harmful eating behaviors [31,35]. Finally, problematic social media use was correlated with negative body image among women only, reflecting their greater engagement in appearance comparisons [78] and societal pressures [79,80], which may lead to risky body modification attempts [81].

### 4.2. Differences Between Research Groups

Hypothesis 2 regarding age group differences was fully confirmed. Emerging adults (18–25) reported higher problematic social media use, internalization of appearance ideals, negative body image, and risk of EDs than young adults (26–40). This is supported by recent studies associating younger age with an increased risk of EDs [33,82,83]. Moreover, emerging adults exhibited lower levels of DoS than young adults, which may reflect developmental differences in emotional regulation and identity consolidation. These differences may be linked to greater reliance on external sources of validation, including appearance-related factors, which have been associated with increased vulnerability to EDs.

Hypothesis 3 regarding gender differences was partially confirmed. Women reported higher levels of internalization of appearance ideals, problematic social media use, negative body image, and the risk of EDs, supporting previous research [42,43,84,85]. These gender differences align with objectification theory [29] and the tripartite influence model [5], which highlight how Western societies prioritize physical appearance in defining women’s values and identity [86]. This pressure is reflected in gendered appearance ideals, with women internalizing and feeling pressured to conform to the Western beauty standard of thinness, while men internalize and feel pressured by ideals associated with a muscular and athletic physique [31,32].

In terms of DoS dimensions, women reported higher levels of emotional reactivity/fusion with others, likely reflecting societal expectations for women to be more emotionally expressive and relationship-oriented [87]. Contrary to expectations, men did not report higher emotional cutoff or I-position than women. This unexpected finding, particularly regarding emotional cutoff, may reflect shifting norms in Western societies, where men’s emotional expression is increasingly accepted [88].

In addition, the interaction analyses pointed to more nuanced gender-specific patterns. Problematic social media use was associated with more negative body image among males, but not among females. One possible interpretation is that, among females, negative body image may already be embedded within a broader and more pervasive pattern of appearance-related pressures, internalization, and self-objectification, such that problematic social media use does not emerge as a uniquely distinguishing correlate. Among males, by contrast, problematic social media use may represent a more specific correlate of body-related self-evaluation, particularly in relation to the increasing visibility of muscular and athletic appearance ideals in social media environments [31,32]. In this context, increased exposure to muscularity-focused content, together with processes of social comparison and external validation, may intensify body-related preoccupation and heighten sensitivity to appearance-based self-evaluation among men. Moreover, although negative body image was associated with greater ED risk in both genders, this association was stronger among females. This finding is consistent with the broader literature and with objectification theory, which emphasize the central role of body dissatisfaction in women’s appearance-related pressures and eating-related vulnerability. However, recent findings suggest that sensitivity to appearance-related pressures is also increasing among men, although it may manifest in different patterns, such as a greater emphasis on muscularity rather than thinness [29,42,43,84,85,86].

Hypothesis 4 regarding clinical differences was partially confirmed. Associations were observed between the study variables and the clinical risk of EDs, with those at risk of EDs reporting higher internalization of appearance ideals, greater emotional reactivity/fusion with others, lower I-position, and lower body image, aligning with previous research [4,7,31]. These findings underscore each factor’s independent association with the risk of EDs, beyond the mediation model. However, contrary to our hypothesis, problematic social media use was not directly associated with clinical ED risk. This finding suggests that the relationship between social media use and ED risk may follow a more complex pathway than initially hypothesized, possibly operating through mediating factors rather than through direct association.

Interestingly, only 15% of participants fell within the clinical range for EDs, lower than previous findings in Israel, such as a recent study reporting 28.5% [8] This lower rate is notable given that data collection occurred during the Iron Swords war that began in October 2023. While the risk of EDs often rises during crises [89,90], the protective role of social solidarity during national crises may serve as a buffer against some of the factors that typically contribute to heightened risk, offering a potential explanation for this result [91,92].

### 4.3. Demographic Characteristics

The examination of demographic characteristics revealed associations between the study variables and participants’ marital status, education, and economic status. Specifically, being single and child-free was linked to higher levels of the research variables and lower DoS, aligning with Bowen’s family systems theory [9], which suggests that low DoS may hinder intimate relationships. Lower educational and economic status correlated with higher research variables (except DoS). Although EDs have traditionally been associated with higher economic status, they are now prevalent across all socioeconomic populations [43], with increased risk in lower socioeconomic groups attributed to reduced health education [91] and inequality in access to resources [93]. Lastly, BMI was positively associated with higher internalization of appearance ideals and negative body image, suggesting that higher BMI may be linked to appearance-related pressures and body dissatisfaction [75].

### 4.4. Profile of Participants at Risk of EDs

The profile of individuals at greater risk of EDs includes females, younger individuals, and those who are unmarried, child-free, and from lower academic and socioeconomic backgrounds. Gender-specific differences are evident: for females, the profile includes lower DoS, higher internalization of appearance ideals, more problematic social media use, a poorer body image, and greater societal pressures related to appearance. For males, the profile includes lower DoS, higher emotional cutoff, greater internalization of muscularity ideals, and more problematic social use media.

## 5. Limitations

This study, while offering valuable insights, is not without limitations. First, it is based on self-reports, which may be subject to social desirability bias. Second, the quantitative nature of the study could benefit from mixed-methods approaches, including in-depth interviews to capture richer data about participants’ experiences. Third, data collection during the Iron Swords war (2024) may have introduced confounding variables, particularly regarding problematic social media use, which tends to increase during stressful periods [39]. Fourth, the limited number of male participants restricted gender-based mediation analyses, highlighting the need for larger, more balanced samples in future research. Fifth, the Israeli sample, with an overrepresentation of participants from lower socioeconomic backgrounds—possibly due to the young sample still being in career transition—may limit generalizability. Sixth, certain methodological aspects warrant consideration, such as the cross-sectional design, which limits causal inference, and the fact that, although almost all measures demonstrated good internal consistency, the factorial structure of the Hebrew versions of the SATAQ-4 and BSQ-C8 was not formally examined within the present sample. Future research should further validate these Hebrew versions. Seventh, the use of the EAT-26 as a three-factor measure, with a threshold score of 20 (see [69]), was chosen because the subscales were highly intercorrelated and it was important for us to examine the clinical cutoff score as an outcome. In addition, the oral control subscale showed relatively lower reliability than the other EAT-26 subscales, which should be taken into account when interpreting findings related to this dimension. In addition, although the SATAQ-4 subscales were positively intercorrelated and showed high overall internal consistency, aggregating them into a single composite measure may have reduced conceptual specificity, particularly with regard to different sources of appearance-related pressure and different appearance ideals. Eighth, gender differences were examined through separate regressions and comparative analyses rather than a multiple-group path model. We opted for the latter approach due to sample size and distribution constraints, which reduced statistical power for multi-group SEM, while still allowing for the identification of meaningful gender differences with stable and interpretable results. Ninth, while problematic social media use was a significant mediator, only 10% of participants fell in the clinical range. Future research should target heavy social media users, include a larger, more representative sample, and replicate the study during periods of peace to isolate the effects of social media use. Finally, the reliance on convenience sampling may introduce selection bias and limit the generalizability of the findings.

***Contributions***

*Notwithstanding* the aforementioned limitations, this study makes significant contributions. Theoretically, it offers a comprehensive framework for understanding the complex factors underlying the risk of EDs through three main contributions. First, the validated mediation model demonstrates how family patterns (DoS) contribute to the risk of EDs through problematic social media use, internalization of appearance ideals, and negative body image. Second, the study identifies distinct patterns across different groups, revealing heightened risk among emerging adults compared to young adults, specific gender-based manifestations, and meaningful differences between individuals meeting clinical criteria for EDs and those who do not. Third, by integrating these findings, the study provides a comprehensive profile identifying individuals most vulnerable to developing EDs. Methodologically, the Hebrew versions of the SATAQ-4 and BSQ-C8 were validated, providing valuable tools for Israeli researchers and clinicians.

***Practical Contributions***

From a practical perspective, the current findings, while preliminary and based on cross-sectional data, offer a tentative foundation for identifying individuals at greater risk for EDs and for informing prevention and intervention efforts. In particular, psychologists, family therapists, and educational counselors may consider drawing on these insights to inform the development of research-based workshops, training programs, and individual or group interventions. Such efforts could focus especially on populations that may be at elevated risk, particularly emerging adults, women, and individuals from lower socioeconomic and educational backgrounds. In addition, interventions may benefit from addressing not only eating-related symptoms but also associated vulnerability factors identified in the present study, namely problematic social media use, internalization of appearance ideals, negative body image, and lower DoS. Where appropriate, special attention could be given to strengthening DoS, particularly in relation to emotional reactivity, fusion with others and I-position, given their observed associations with greater susceptibility to appearance pressures and ED risk. Finally, prevention efforts may also consider fostering more critical engagement with appearance-related social media content and promoting healthier body image, while recognizing that these implications should be interpreted with caution due to the study’s design limitations.

## Figures and Tables

**Table 1:** Means, standard deviations, and correlations of the study variables (*N* = 333).

	*M* (*SD*)	1.	2.	3.	4.	5.	6.
1. DoS: emotional reactivity/fusion with others	3.42 (0.93)						
2. DoS: emotional cutoff	2.56 (0.88)	0.57 ***					
3. DoS: I-position	3.65 (0.90)	−0.15 **	−0.07				
4. Problematic social media use	2.79 (2.09)	0.35 ***	0.28 ***	−0.18 ***			
5. Internalization of appearance ideals	2.27 (0.75)	0.26 ***	0.25 ***	−0.20 ***	0.28 ***		
6. Negative body image	89.36 (43.70)	0.34 ***	0.31 ***	−0.23 ***	0.24 ***	0.65 ***	
7. Risk of EDs	10.28 (11.02)	0.36 ***	0.28 ***	−0.12 *	0.13 *	0.49 ***	0.54 ***

* *p* < 0.05, ** *p* < 0.01, *** *p* < 0.001. Note. Ranges: DoS 1–6; problematic social media use 0–9; internalization of appearance ideals 1–5; negative body image 34–204; risk of EDs 0–130.

**Table 2:** Means, standard deviations, *t* values, and Pearson correlations for the internalization of appearance ideals by gender (*N* = 333).

	Total *M* (*SD*)	Males *M* (*SD*)	Females *M* (*SD*)	*t*(*df*) (*p*)	DoS: Emotional Reactivity/Fusion with Others	Dos: Emotional Cutoff	DoS: I-Position	Problematic Social Media use	Negative Body Image	Risk of EDs
Internalization of the thin/low body fat ideal	2.75 (1.10)	2.49 (0.95)	2.91 (1.15)	*t*(302.83) = 3.60 (*p* < 0.001)	0.23 ***	0.18 **	−0.21 ***	0.14 *	0.66***	0.54 ***
Internalization of the athletic/muscular ideal	2.41 (1.09)	2.56 (1.08)	2.31 (1.09)	*t*(331) = −2.07 (*p* = 0.039)	−0.04	0.06	−0.06	0.10	0.17 **	0.26 ***
Family pressure	2.11 (1.13)	2.03 (1.02)	2.16 (1.20)	*t*(295.36) = 1.05 (*p* = 0.295)	0.23 ***	0.24 ***	−0.07	0.25 ***	0.44 ***	0.30 ***
Peer pressure	1.49 (0.80)	1.63 (0.88)	1.39 (0.74)	*t*(230.90) = −2.56 (*p* = 0.011)	0.07	0.18 **	−0.10	0.22***	0.30 ***	0.12 *
Media pressure	2.46 (1.40)	1.77 (1.05)	2.89 (1.42)	*t*(317.93) = 8.26 (*p* < 0.001)	0.36 ***	0.21 ***	−0.22 ***	0.26 ***	0.56 ***	0.35 ***

* *p* < 0.05, ** *p* < 0.01, *** *p* < 0.001. Note. Ranges: DoS 1–6; problematic social media use 0–9; internalization of appearance ideals 1–5; negative body image 34–204; risk of EDs 0–130.

**Table 3:** Indirect paths for the serial mediation regarding the risk of EDs (*N* = 333).

Independent Variables	Mediators	Indirect Effect	*SE*	*p*	95%*CI*
DoS: emotional reactivity/fusion with others	Problematic social media use ⟶ Internalization of appearance ideals ⟶ Negative body image	0.008	0.004	<0.001	0.003, 0.018
DoS: emotional cutoff	Problematic social media use ⟶ Internalization of appearance ideals ⟶ Negative body image	0.004	0.003	0.011	0.001, 0.012
DoS: I-position	Problematic social media use ⟶ Internalization of appearance ideals ⟶ Negative body image	−0.004	0.002	0.003	−0.011, −0.001
Problematic social media use	Internalization of appearance ideals ⟶ Negative body image	0.030	0.012	0.001	0.012, 0.058
Internalization of appearance ideals	Negative body image	0.176	0.035	<0.001	0.113, 0.253
DoS: emotional cutoff	Internalization of appearance ideals ⟶ Negative body image	0.024	0.012	0.018	0.005, 0.054
DoS: I-position	Internalization of appearance ideals ⟶ Negative body image	−0.025	0.011	0.004	−0.051, −0.008
DoS: emotional reactivity/fusion with others	Negative body image	0.044	0.018	0.002	0.015, 0.084
DoS: emotional reactivity/fusion with others	Problematic social media use ⟶ Internalization of appearance ideals	0.011	0.005	<0.001	0.004, 0.025
DoS: emotional cutoff	Problematic social media use ⟶ Internalization of appearance ideals	0.006	0.004	0.011	0.001, 0.016
DoS: I-position	Problematic social media use ⟶ Internalization of appearance ideals	−0.006	0.003	0.003	−0.015, −0.001
DoS: emotional cutoff	Internalization of appearance ideals	0.033	0.017	0.017	0.006, 0.076
DoS: I-position	Internalization of appearance ideals	−0.034	0.015	0.004	−0.071, −0.011
Problematic social media use	Internalization of appearance ideals	0.041	0.016	<0.001	0.015, 0.081

**Table 4:** Logistic regressions for the classification of the risk of EDs, with the study variables (*N* = 333).

	*OR*	*p*	95%*CI*
DoS: emotional reactivity/fusion with others	1.54	0.031	1.04, 2.28
DoS: emotional cutoff	1.41	0.051	0.99, 2.00
DoS: I-position	0.60	0.007	0.42, 0.87
Problematic social media use	0.97	0.704	0.82, 1.14
Internalization of appearance ideals	5.67	<0.001	3.27, 9.82
Negative body image	111.97	<0.001	28.83, 434.82
Internalization of appearance ideals subscales:			
Internalization of the thin/low body fat ideal	4.56	<0.001	2.93, 7.10
Internalization of the athletic/muscular ideal	1.80	<0.001	1.33, 2.45
Family pressure	3.73	<0.001	1.87, 7.45
Peer pressure	3.29	0.049	1.01, 10.77
Media pressure	1.74	<0.001	1.33, 2.26

## Data Availability

The data presented in this study are available from the corresponding author upon reasonable request.
